# Static stretching does not alter pre and post-landing muscle activation

**DOI:** 10.1186/1758-2555-3-9

**Published:** 2011-05-13

**Authors:** Wesley R Moss, J Brent Feland, Iain Hunter, J Ty Hopkins

**Affiliations:** 1 Human Performance Research Center, Brigham Young University, Provo, UT

## Abstract

**Background:**

Static stretching may result in various strength and power deficiencies. Prior research has not determined, however, if static stretching causes a change in muscle activation during a functional task requiring dynamic stability. The purpose of this study was to determine if static stretching has an effect on mean pre and postlanding muscle (vastus medialis VM, vastus lateralis VL, medial hamstring MH, and biceps femoris BF) activity.

**Methods:**

26 healthy, physically active subjects were recruited, from which 13 completed a 14-day static stretching regimen for the quadriceps and hamstrings. Using the data from the force plate and EMG readings, a mean of EMG amplitude was calculated for 150 msec before and after landing. Each trial was normalized to an isometric reference position. Means were calculated for the VM, VL, MH, and BF from 5 trials in each session. Measures were collected pre, immediately following the 1^st ^stretching session, and following 2 weeks of stretching.

**Results:**

A 14-day static stretching regimen resulted in no significant differences in pre or postlanding mean EMG amplitude during a drop landing either acutely or over a 14-day period.

**Conclusions:**

Static stretching, done acutely or over a 14-day period does not result in measurable differences of mean EMG amplitude during a drop landing. Static stretching may not impede dynamic stability of joints about which stretched muscles cross.

## Introduction

Stabilizing muscle contractions are very common during sports movements. Contraction of muscles crossing a joint provides stiffness in addition to controlling movement. This stiffness, which provides additional support to mechanical or inert stabilizers, is referred to as dynamic stability[1]. Dynamic stability has been measured in the past by quantifying proprioception,[2,3] cutaneous sensation,[4,5] nerve conduction velocity,[4,6] neuromuscular response time,[7-9] postural control,[10-12] and strength[13,14]. The sensorimotor system has a major role in dynamic stability, which may be compromised by any impairment of neuromuscular function.

Throughout sports, static stretching is commonly used prior to activity. However, pre-event stretching may not be beneficial to performance[15-18]. An evident decrease in muscle function has been shown immediately following prolonged passive, static stretching[19,20]. Avela et al[19]. reported a reduction of reflex sensitivity of short-latency reflexes due to prolonged, passive, static muscle stretching. Further, Herda and colleagues [20] reported that prolonged, passive stretching (135 s bouts repeated over 20 min) along with tendon vibration results in diminished force production, suggesting that the stretching stimulus could cause gamma loop dysfunction. In fact, static stretching has resulted in deficits in force production lasting up to 120 minutes[21]. Additionally, a decrease in gamma motoneuron (MN) activation and spindle sensitivity could result in alterations to stiffness of muscles crossing joints and dynamic stability during sports activities [22]. Significantly altered spindle sensitivity would result in changes to muscle latency and amplitude during a demanding, functional task as the sensorimotor system works to stiffen the joint through contraction.

It is unclear whether static stretching delays and/or decreases muscle activation during functional sports movements. More specifically, it is unclear whether common stretching loads and durations (as opposed to prolonged stretching loads) used by athletes and sports medicine professionals would result muscle activation alterations that would affect contractions during a stabilizing activity. Many sports utilize jumping and landing along with the dynamic stability necessary to protect the joint, and therefore a drop landing could be seen as a functional task associated with dynamic stability. The purpose of this study was to determine if clinically relevant acute and chronic static stretching caused alterations in mean electromyographic (EMG) amplitude of the surrounding muscles of the knee (vastus lateralis, vastus medialis, biceps femoris, and medial hamstrings) during a drop landing.

## Methods

Twenty-six volunteers were recruited for participation in this study. Fourteen females (age, 22 ± 2 years, height, 1.68 m ± .05 m, weight, 61.95 kg ± 10 kg) and 12 males (age, 25 ± 3 years, height, 1.79 m ± .05 m, weight, 78.11 kg ± 8.8 kg) all signed an informed consent that was approved by the Institutional Review Board. Written informed consent was obtained from the patient for publication of this research and accompanying images. A copy of the written consent is available for review by the Editor-in-Chief of this journal. Volunteers were free from injury to the lower extremity at least three months prior to the study, did not exceed normal limits of hamstring and quadriceps flexibility, and were physically active, partaking in regular physical activity three times per week for at least 20 minutes a session. Subjects were randomly assigned to an intervention group (n = 13) or a control group (n = 13). One subject dropped out of the study due to an injury of the ankle two days prior to final testing. Two other subjects were dropped due to instrumentation errors.

The examiner lightly abraded and cleaned areas on the dominant leg for EMG electrode placement on the vastus medialis, vastus lateralis, biceps femoris, and medial hamstrings. Electrodes were placed 2 cm center to center and in line with the longitudinal axis of the muscle. A ground electrode was placed on the head of the fibula. Electrode placement was visually verified inspecting the signal during an isometric contraction. Electrodes were outlined with permanent marker for replacement during subsequent data collection sessions. EMG data were recorded using a Biopac MP150 system (BIOPAC Systems Inc., Santa Barbara, CA). Signals were amplified (TEL100M, BIOPAC Systems Inc., Santa Barbara, CA) from disposable, pre-gelled Ag-AgCl electrodes. The input impedance of the amplifier was 1.0 megaohm, with a common mode rejection ratio of 110 dB, high and low pass filters of 10 and 500 Hz, a signal to noise ratio of 70 dB, and a gain of 1000. EMG data were collected at 2000 Hz using Acknowledge 3.73 software package (BIOPAC Systems Inc., Santa Barbara, CA). EMG data were processed using a root mean square (RMS) algorithm with a 10 ms moving window.

With each testing session, the subjects maintained an isometric reference position (IRP) lasting 15 seconds in order to serve as a normalization contraction for EMG amplitude measurements. Athletic tape was used on the floor to mark shoulder width foot position. A 45-pound bar was balanced across the posterior neck and shoulders, very similar to the typical technique for squatting exercises. A goniometer was utilized to ensure both knees were positioned with a joint angle of 30 degrees of flexion. Subjects were instructed to balance in this position and keep the weight of the bar equally distributed across both feet. Subjects performed three repetitions prior to baseline and final testing. These data were filtered and processed as previously described and a mean value from a 150 msec window between 7 and 8 seconds was taken for normalization of each landing trial.

Subjects performed 3-5 practice drop landings from a platform (47 cm) onto the force plate (AMTI OR6, Newton, MA). The subjects were instructed to hang the dominant leg off the platform so they were able to "fall" onto the force plate, landing on the dominant leg. The subjects were instructed not to jump off the platform, and to land as if they were landing from a jump. Testing sessions included 7 drops with 30 seconds rest observed between each trial. Sessions were completed prior to any flexibility intervention, immediately following the first flexibility session, and following 14 days of flexibility intervention. Data from the force plate provided the onset of landing for data reduction. These data were input into the EMG capture for a time reference.

The hamstring flexibility measurement technique used in this study was the lying passive knee-extension test (LPKE)[23]. A brief description of this method is as follows:

When performing the LPKE, the subjects lay supine on an examination table. A mark (made with a permanent marker) was made on the lateral leg (about the level of the knee), which corresponded to a mark on the table. This mark was to assure positioning of the subjects when performing the follow-up measurement. The non-dominant leg stayed positioned against the table, while the dominant leg, which was measured in each test, was positioned at 90 degrees of hip flexion so that the thigh rested against a cross-bar that was attached to the table to allow consistent positioning of each repeated hip flexion. The subject was asked to "relax" as much as possible and the examiner then passively moved the tibia into terminal knee extension, which was operationally defined as the point at which the subject began to complain of "mild discomfort" in the hamstrings. At this point the knee-extension value (as measured using and inclinometer) was recorded. The inclinometer was placed just below the tibial tuberosity.

Subjects in the control group were not required to do anything but continue activities of daily living and test at the same time as subjects in the stretching group. Subjects in the stretching group, were instructed to perform five thirty-second bouts of static stretching of the hamstrings and quadriceps. Stretching of each muscle group lasted no longer than five minutes. The entire stretching treatment lasted a total of ten minutes each day for 14 continuous days. All weekday sessions were supervised to ensure consistency. On weekends, the tester notified the subjects by phone to remind them to stretch on their own. In reply the subjects notified the tester once the regimen was completed.

**Figure 1:**
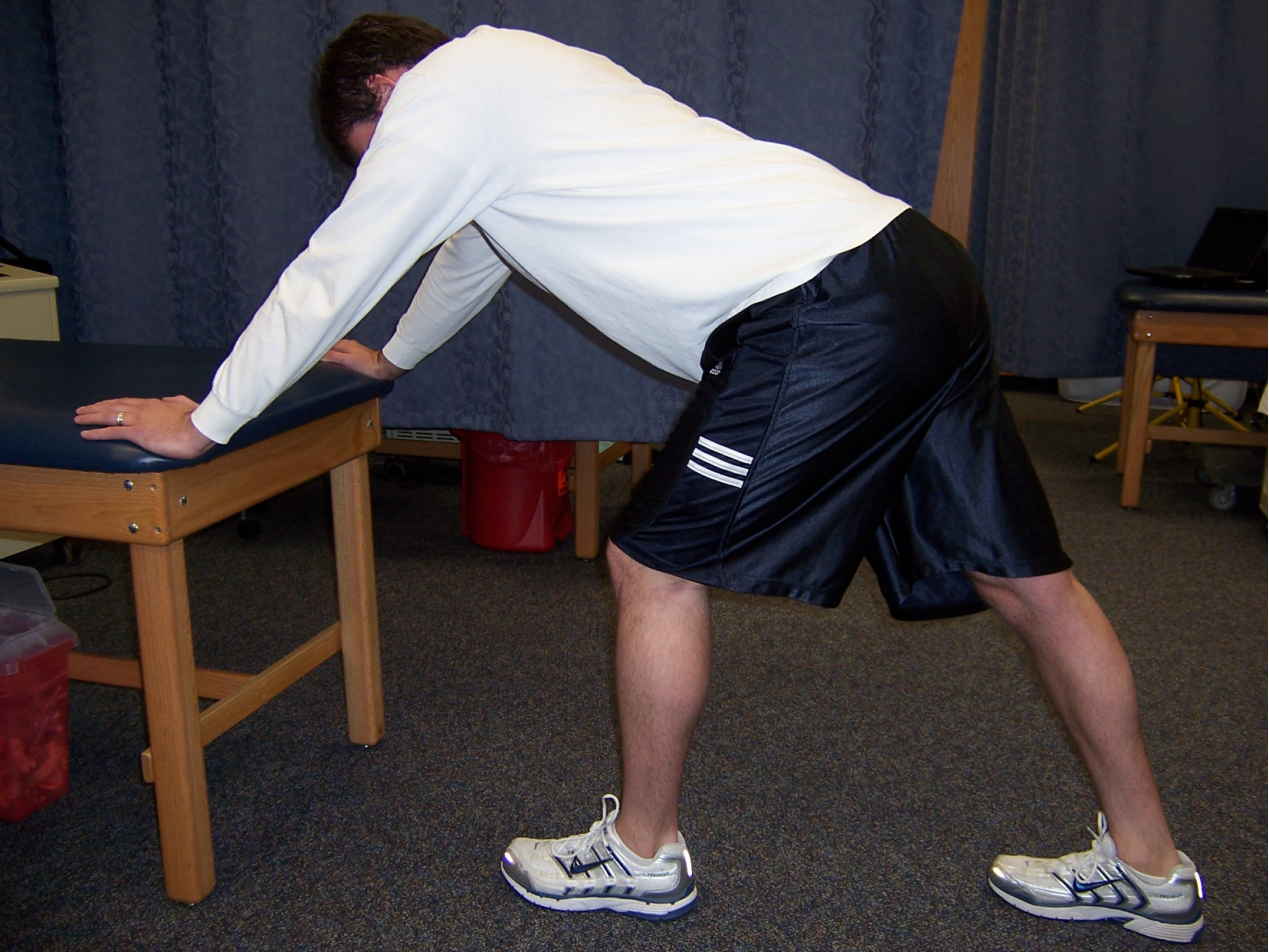
**Position of the subject during static stretching of the hamstrings**. The left limb is being stretched.

The dominant leg of each subject was stretched from a standing position. The subject positioned him/herself near a table for aid in stabilization during stretching. Each subject positioned him/herself with the treatment leg approximately two feet in front of the opposite leg, and brought the chest toward the knee, while keeping their back straight and using the table for balance and to minimize stabilizing contraction of the hamstrings (Figure 1). In order to ensure a sufficient stretch, the subject kept the leg being stretched in a straight position and bent forward taking each stretch to a point of slight discomfort and holding that position.

**Figure 2:**
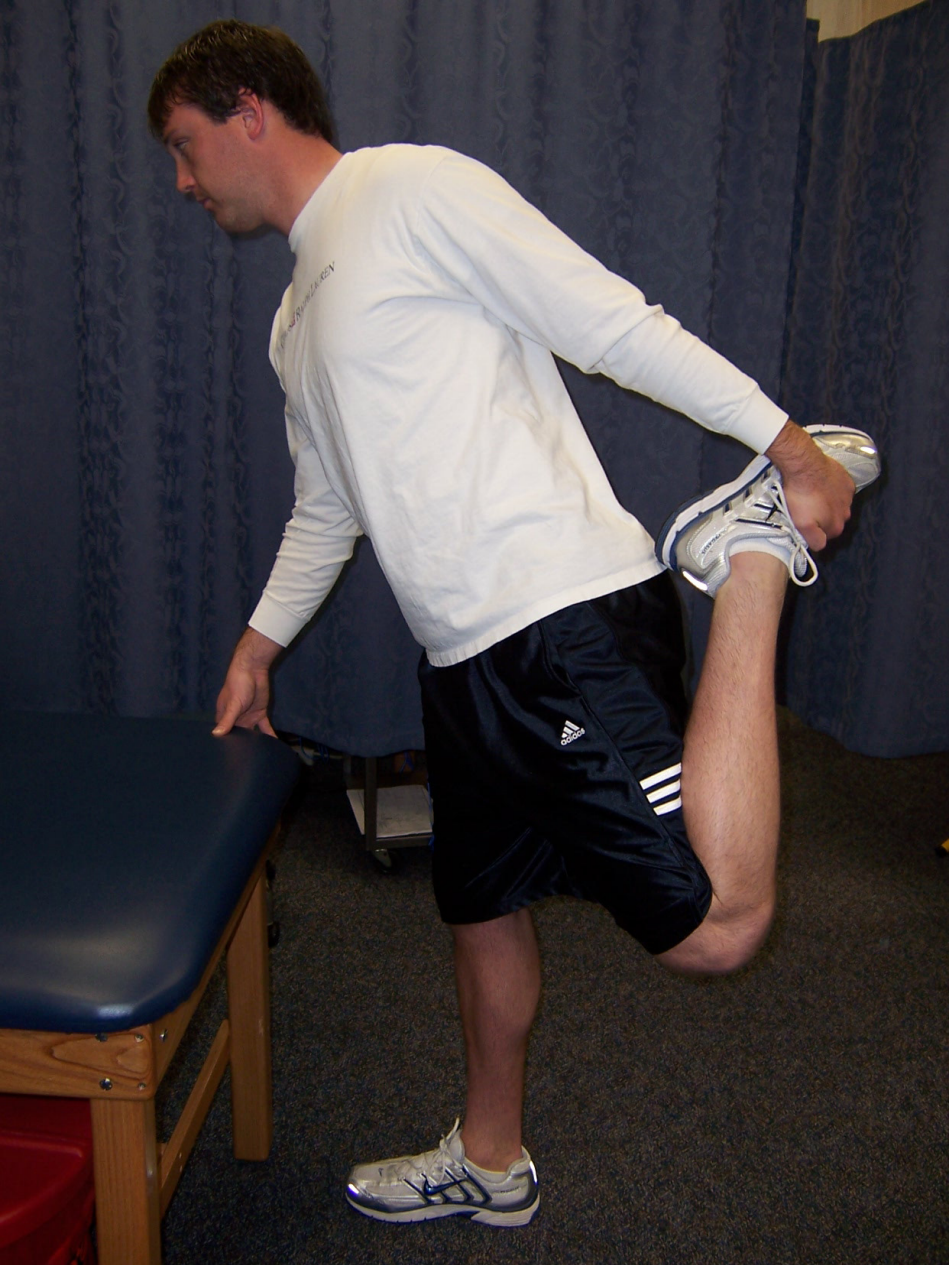
**Position of the subject during static stretching of the quadriceps**.

The quadriceps stretch required the subject to stand with the aid of a table or chair for stability. The subject grasped the ankle of the leg being stretched with the ipsilateral hand, and the knee was flexed so that the heel of the foot approached the buttocks (Figure 2)[24]. This stretch of the knee extensors was taken to a point of slight discomfort and held in that position. All stretches were repeated five times for 30 seconds each. Five to ten seconds rest were observed between each stretch.

Prior to testing, hamstring flexibility was measured for all subjects. A total of three testing sessions were completed for the entire experiment. For the first testing session, every subject, regardless of group, performed seven pretreatment drop landing trials to attain baseline measurements on the first day. A period of approximately 30 seconds elapsed between each trial while the subject was reset. Pre and postlanding EMG amplitudes were measured for the vastus lateralis, vastus medialis, medial hamstrings, and lateral hamstrings. All testing sessions were completed in this fashion. Subjects in the treatment group began their static stretching regimen immediately following the pre-treatment trials. Subjects in the control group rested for 10 minutes. The second testing session took place immediately after completion of either the 10-minute stretching regimen for the stretching group or ten minutes of rest for the control group. After these acute posttreatment measurements were recorded, the subjects in the treatment group continued their static stretching regimen for a total of 14 days, while the subjects in the control group continued their normal activities for the duration of the experiment. On day fourteen, all subjects were again tested in the same fashion.

EMG amplitude values prior to (150 ms) and following (150 ms) landing from the vastus lateralis, vastus medialis, medial hamstrings, and biceps femoris were collected from 26 subjects for analysis. Each value was normalized to the mean IRP for that measurement session. High and low values for each session were dropped and means of the remaining 5 trials were analyzed using a 2 × 3 ANOVA with repeated measures on time to detect differences between groups over time. A 2 × 2 ANOVA with repeated measures on time was used to determine if knee extension with 90° of hip flexion measures were different between pretreatment and final posttreatment between groups. Statistical significance was set at *p *= 0.05.

## Results

Tables 1 and 2 provide summary data for mean EMG activation prelanding and postlanding. No prelanding effect was observed between groups (control and stretch) over time (pretreatment, acute posttreatment, and final posttreatment) for the VL (F_(2,48) _= 0.560, *P *= .575), VM (F_(2,48) _= 0.083, *P *= .921), MH (F_(2,48) _= 0.935, *P *= .400), or BF (F_(2,48) _= 0.585, *P *= .561). No group by time postlanding interaction was detected for the VL (F_(2,48) _= 0.244, *P *= .784), VM (F_(2,48) _= 0.147, *P *= .864), MH (F_(2,48) _= 0.262, *P *= .770), or BF (F_(2,48) _= 1.776, *P *= .180). Knee extension values for the final posttreatment period with the hip in 90° of flexion were analyzed to ensure an appropriate stretching load. A group by time interaction was detected (F_(1,24) _= 27.90, *P *= 0.0001) with the stretch group increasing in ROM by 7.31° (*P *< 0.05) and the control group displaying no change in ROM (-2.54°).

**Table 1:** Prelanding Normalized Mean EMG Amplitude (Mean ± SD).

	Vastus Lateralis		Vastus Medialis		Medial Hamstrings		Biceps Femoris	
	Stretch	Control	Stretch	Control	Stretch	Control	Stretch	Control
Pretreatment	.94 ± .44	1.08 ± .52	1.21 ± .48	1.35 ± .70	6.25 ± 4.83	6.03 ± 3.96	4.63 ± 4.05	3.86 ± 1.78
Acute Posttreatment	.92 ± .47	1.59 ± 1.81	1.25 ± .55	1.32 ± .64	6.14 ± 4.36	5.99 ± 4.61	4.56 ± 3.95	4.06 ± 1.64
Final posttreatment	1.14 ± .52	1.64 ± .98	1.42 ± .58	1.52 ± .82	8.02 ± 4.35	6.45 ± 5.52	5.47 ± 3.99	5.79 ± 4.58

**Table 2:** Postlanding Normalized Mean EMG Amplitudes (Mean ± SD).

	vastus lateralis		vastus medialis		medial hamstrings		biceps femoris	
	Stretch	Control	Stretch	Control	Stretch	Control	Stretch	Control
Pretreatment	4.56 ± 1.63	5.57 ± 3.01	3.73 ± 1.39	4.61 ± 2.72	7.83 ± 4.07	6.73 ± 2.50	9.78 ± 9.75	6.11 ± 3.03
Acute Posttreatment	4.69 ± 2.29	6.95 ± 5.21	3.91 ± 1.66	4.56 ± 2.10	8.61 ± 6.57	6.24 ± 2.63	7.90 ± 6.91	6.04 ± 2.48
Final posttreatment	4.05 ± 3.82	5.57 ± 4.34	3.03 ± 2.01	3.80 ± 3.73	5.55 ± 2.55	5.31 ± 4.47	4.58 ± 3.41	8.61 ± 9.79

## Discussion

This study did not support the hypothesis that static stretching of the quadriceps and hamstrings will suppress dynamic stabilizing contractions of the knee. This was demonstrated by EMG amplitudes following one session of static stretching and following 14 continuous days of static stretching.

Although there are no previous studies that involve muscle activation during a drop landing relative to static stretching, there are studies that report the effects of static stretching on muscle function. One study theorized that prolonged, repeated passive stretching lead to a diminished stretch response as a result of increased muscle tissue compliance[19]. However, it should be noted that the stretch used in the previously mentioned study lasted for 60 min. Herda and colleagues [20] also found that a prolonged passive stretch (135 s stretches repeated over 20 min) caused similar force decrements as tendon vibration. They speculated that such decrements could be driven by changes to the gamma loop, supporting the ideas proposed by Avela et al. Cramer et al. reported static stretching to decrease a muscle's ability to produce force at fast and slow velocities[24]. It was also reported that maximal strength deficits were found following static stretching[25]. These studies represent a compromise in muscle force generation, but others have reported no change[26,27]. While the literature has reported deficits in muscle function following static stretching, [17-19,21,24,25] this study attempted to determine if these deficiencies translate into deficits in muscle activation during a stabilizing contraction. Further we used a stretching protocol similar in duration to those used clinically and with athletes. While stretching stimuli of differing magnitudes, speeds, and durations have resulted in muscle function deficits, our results suggest that muscle activation immediately prior to and following a drop landing is not affected by a common static stretching protocol.

The fact that no significant differences were found between groups is an indication that static stretching, acutely, or when done for 14 days, has no effect on preparatory or reactive muscle activity during a drop landing. Certain clinical implications can be supported by these data. As mentioned before, it was reported that diminished stretch responses came as a result of prolonged static stretching and its increased effects on muscle tissue compliance[19]. This information is significant when dealing with the dynamic action of a drop landing. According to the results of this study, it may be implied that static stretching will not suppress the amplitude of short latency responses that contribute to stabilizing contraction following a drop landing. While the stretching protocol was very different between studies, Avela et al. [19] suggested that prolonged, static stretching negatively affects the spindle and resultant reflexive activation. However, these reflexes could be modified by the large stimulus associated with dynamic, high load actions like a drop landing, suggesting that static stretching may not negatively affect short latency responses associated with stabilizing the joint during these types of actions. In other words, perhaps the load and stretch associated with a drop landing is high enough to reach muscle spindle threshold, even if the sensitivity has been reduced. Further data are needed to determine if muscle latencies might be negatively affected. Anticipation could also play a large role in pre and postlanding activation. While no difference in prelanding or preparatory muscle activation was detected between groups at any given time, landing anticipation could have played a significant role in masking any effect from static stretching. However, one might assume that this same anticipation would exist in many competitive environments wherein these athletes compete. Given the data from this study, static stretching did not appear to negatively affect the motor strategies associated with stabilizing muscle activation during a drop landing.

While care was taken to control for many variables in this study, limitations should be considered. Subjects were asked to take the stretches to a point of slight discomfort and hold that position for 30 seconds. The intensity of the stretch may have been different from subject to subject due to independent differences in pain tolerance or perception. Also, 30 seconds is considered an optimal duration for static stretching,[28] but 14 days may not have been a long enough period of time to detect any significant changes in muscle activity. However, despite these possible differences in stretching intensity and duration, all but one of the subjects in the stretching group displayed gains in ROM, which verified that the stretches were effective in increasing range of motion. Each of the subjects in the stretch group displayed average gains in ROM of 7.3°± 5.8°, while subjects in the control group displayed average deficits of -2.5°± 3.3°. Hamstring flexibility was monitored to reflect that the flexibility protocol used in this study was sufficient in order to see increases in ROM. Also, due to the difference in body types, landing biomechanics may have differed due to longer limbs, different posture, and contrasting landing styles. In other words, one uniform landing height might have resulted in varied landing mechanics. However, the mean height between groups was matched well and this likely had little affect on our results. Finally, it should be noted that a drop landing was used in this study as a representative movement that requires high levels of stabilizing contraction. However, these muscles also contracted to control movement, and EMG is a non-discriminate measure of muscle activation.

## Conclusion

Our findings revealed no significant differences in pre and postlanding EMG amplitude between the control group and stretching group. These results were constant across baseline, acute posttreatment, and final posttreatment measurements. These findings are unique in that no other authors, to our knowledge, have examined the effects of static stretching on muscle activation prior to and following a functional stabilization task common to sports. These data may imply that static stretching will not suppress dynamic stability during a drop landing.

## Competing interests

None of the authors have received reimbursements, fees, funding, or salary from an organization that may in any way gain or lose financially from the publication of this manuscript. There are no other competing interests of any kind associated with the publication of these data.

## Authors' contributions

All authors 1) have made substantial contributions to conception and design, or acquisition of data, or analysis and interpretation of data; 2) have been involved in drafting the manuscript or revising it critically for important intellectual content; and 3) have given final approval of the version to be published.
